# Resurrecting FUS: Adrenal Androgens as an Ultimate Cause of Hematuria, Periuria, Pollakuria, Stranguria, Urolithiasis and Obstruction in Neutered Cats

**DOI:** 10.3389/fvets.2018.00207

**Published:** 2018-09-11

**Authors:** Brandon P. Reines, Robert A. Wagner

**Affiliations:** ^1^Department of Biomedical Informatics, University of Pittsburgh School of Medicine, Pittsburgh, PA, United States; ^2^St. Francis Animal Hospital, North Huntingdon, PA, United States; ^3^Department of Laboratory Animal Resources, University of Pittsburgh School of Medicine, Pittsburgh, PA, United States

**Keywords:** cats, animals, androgens, urethral obstruction, urologic diseases, seasons, urinary bladder

## Abstract

Although many authors have doubted that “feline urological syndrome” (FUS) describes a real pathogenetic entity, because it subsumes such a large variety of signs, Sumner's recent finding that urethral obstruction occurs most frequently in springtime adds to a large body of evidence that lower urinary tract problems occur most commonly in late winter and spring. This suggests that FUS may be a unitary disorder, with a hormonal basis, driven by increasing day length. We argue that rising adrenal androgens (AA) in neutered cats induce stress, and other more concrete manifestations of FUS through androgen-driven mechanisms.

## Introduction: ever-changing models of feline urinary disorders

In 1970, Osbaldiston and Taussig analyzed 41 male and 5 female cats suffering from a constellation of urologic problems including hematuria, dysuria, cystitis, urethritis, urolithiasis, and urethral obstruction (1). Those authors argued that the components of the sign complex occurred together often enough to suggest that they comprise a single nosologic and possibly etiologic entity. They named it feline urological syndrome or FUS.

**Figure 1:**
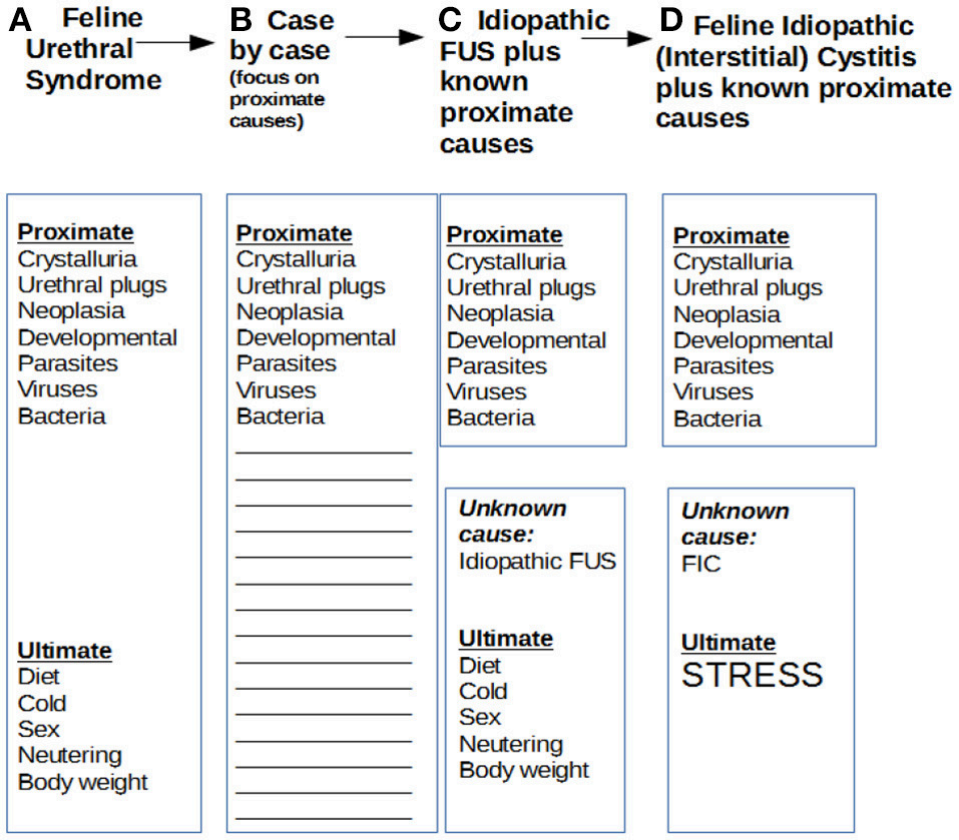
Evolution of models of proximate and ultimate causes of feline urinary disturbance. Over the years, models of feline urinary disturbance, their proximate and ultimate causes, and their designations have changed several times. This has created a confusing situation. In 1970, Osbaldiston and Taussig described cases of irritative voiding with or without obstruction as feline urological syndrome or FUS. In 1984, Osborne's group disagreed that the various cases could be lumped together under a common rubric, and preferred a case by case analysis of proximate causes, which was very generally referred to as feline lower urinary tract disease or FLUTD. A bit later, Barsanti focused also on ultimate causes of FUS, and argued that most cases had not obvious proximate cause, and should be dubbed “idiopathic FUS.” Buffington also focused on idiopathic cases and ultimate causes, the main one he postulated being stress, and shifted the anatomic focus to the bladder by delineating feline interstitial cystitis or FIC.

Although enthusiastically embraced by many veterinarians and researchers for a decade or so, by the early 1980s, the FUS model had acquired an increasing number of critics who contended that FUS was nothing but a sign complex which lacked an adequate pathogenetic basis (2). Such detractors pointed out that nearly half of FUS cases lacked any demonstrable proximate cause such as infection, crystalluria, or urachal remnant to explain irritative voiding, much less complete obstruction. Osborne and co-workers called for a return to a case by case approach to feline lower urinary tract disease generally, where careful diagnostic analysis of each affected patient would define a likely pathogenetic mechanism (see Figure 1) (3).

Partly in reaction to that overfocus on proximate causes, and to attempt to explain the frequent absence of such causes with a deeper if less concrete cause of feline urinary disturbance, Buffington adduced evidence that many such cases are ultimately caused by environmental stress of some kind, a model which became known as feline interstitial or idiopathic cystitis (FIC) (4). This model, although shifting the focus from the urethra to the bladder and highlighting stress, was not very different from Barsanti's idea of “idiopathic FUS” (5).

However, neither Buffington's nor Barsanti's models adequately explain why: (1) the complex of signs and pathological manifestations originally identified as FUS so reliably occur together, (2) why environmental stress induces worst effects on the feline urogenital tract, (3) why FUS and obstruction occur most frequently in late winter and spring, and (4) why neutering of male cats is such a strong risk factor for FUS and obstruction. Such facts implied to us that some other ultimate cause of FUS must exist, and that it is likely to be hormonal in nature. Based on clues from the epidemiology of FUS and related disorders, *we postulate that rising adrenal androgen levels in blood of neutered cats is the most common underlying cause of a cascade of physiological events leading to most of the known manifestations of FUS*.

## Seasonality of FUS: late winter/spring surge in both northern and southern hemispheres implicates an hormonal mechanism

The tendency for FUS and obstructive uropathy to occur during seasons with increasing day length, particularly in late winter and spring, has been highlighted in both small hospital-based studies and large epidemiological studies in both hemispheres including the North American Veterinary Medical Data Program, based on 4,111 cases of FUS, which showed peak incidence in March (6), and, more recently, through analysis of incidence of urethral obstruction alone (7).

It is remarkable that, despite the shifting definitions and categories of feline urinary disturbances over the years, their seasonality has proven consistent in a wide variety of investigations. A few studies show no seasonality (8, 9). But most reveal peak incidence of feline urinary disturbances consisting of hematuria, pollakiuria, stranguria, periuria, and/or obstruction in late winter and spring (10, 11). This is so regardless of hemisphere of the world in which the cats live (12). In the most recent study, Sumner and Rishniw focused solely on urethral obstruction in the United States, and found peak incidence in spring (7). Jones, Sanson and Morris discovered an opposite monthly pattern to the US results in New Zealand, with peak incidence in later months of the year, which corroborates occurrence in late winter/ spring, as those seasons occur in later months of the year in the southern hemisphere (13). In Caston's study, which inspired Buffington's work on feline interstitial cystitis (FIC), cases of hematuria with or without obstruction peaked after an earthquake in California, but also showed higher incidence in late winter and spring (14). In addition, Bernard analyzed cases in Ottawa, Ontario and also detected maximal numbers in late winter/spring (15).

Most authors have interpreted the late winter/spring surge to mean that cold weather is the key causative factor. However, in the Southern hemisphere in warm climates including Brazil (12), the same surge is reliably seen. This suggests that increasing day length *per se* may be the cause of the increased incidence in late winter/spring, directly implicating hormonal mechanisms. In what follows, just which mechanisms are operative is inferred from general knowledge of reproductive physiology of human beings and cats.

## Hypothesis: adrenal androgen (AA) model of feline urologic syndrome

How do we explain the constellation of signs and pathologic manifestations in FUS by reference to rising AA levels in neutered cats? Answering that question requires re-analyzing at least two interrelated bodies of data and theory regarding: (1) interstitial cystitis in women and its presumed neurogenic origin as outlined by Buffington as a model of feline interstitial cystitis (FIC), and (2) possible dual role of AA both as precursors of testosterone and as adaptations to chronic stress. Although Buffington had highlighted that both women with IC and cats with FIC share blunted cortisol responses to ACTH, at the time of his work, no analysis of androgen involvement in women with IC had been undertaken that might provide further clarification.

However, by far the most important new finding in human IC is Parker's identification of a persistent rise in urine levels of the androgen etiocholan-3α-ol-17-one sulfate (Etio-S), a sulfoconjugated 5-β reduced isomer of testosterone, in female IC which distinguishes them from control subjects with a sensitivity and specificity >90% (16). Those authors interpreted their results as outright rejecting the neurogenic view in favor of the notion that the women possess an inherent biochemical abnormality, as Etio-S may drive both neurogenic and inflammatory processes in IC. Adrenal androgens and related neurosteroids (17) are increasingly viewed, however, as part of a system compensating for chronic stress, particularly the detrimental effects of such stress on immunity (18). In conjunction with increasing evidence that androgens, in particular DHEA and DHEA-S, may be part of a stress adaptation syndrome (19), we posit that a similar process may be occurring in a subgroup of our feline patients–those prone to FUS.

**Figure 2:**
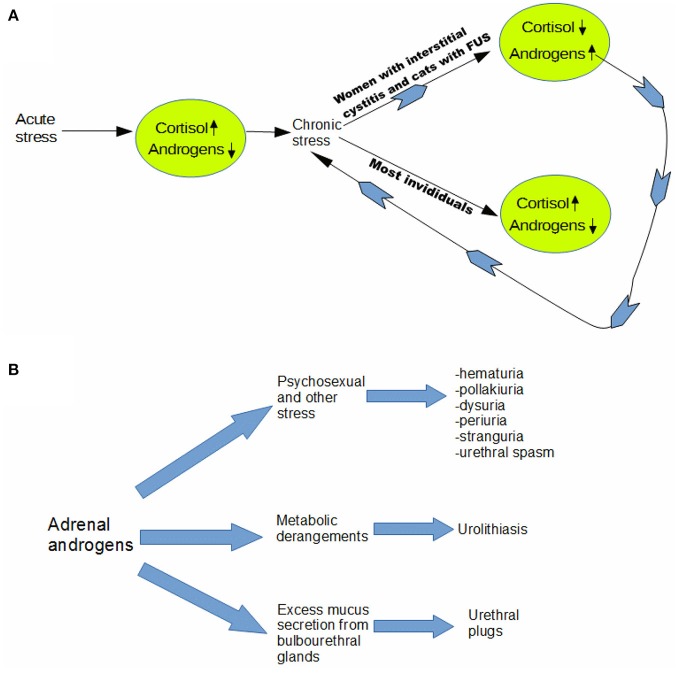
Adrenal androgen model of feline urological syndrome. **(A)** Whether due to stress from external causes or to an endogenous physiological process, we believe that FUS may result as an outcome of the dual role of adrenal androgens as partial agonists of the androgen receptor mediating sexual behavior and as an adaptation to chronic stress in certain feline patients. In that light, rising androgen levels in late winter and spring may constitute a “perfect storm” of factors that lead to an increased incidence of complete urinary obstruction. Androgens may rise not only because of chronic stress in FUS individuals, but breeding season itself may be the underlying stress leading to postulated adaptive response. We contend that at least part of the “stress” experienced by the predisposed cats is psychosexual in origin. Androgens are known to heighten sexual arousal in both males and females (20), and neutering may create a state of suspended arousal, due to inability to complete sex-typical behaviors, due to absence of either testosterone or estrogen. **(B)** We postulate that adrenal androgens are an ultimate cause of most manifestations of feline urologic syndrome (FUS), which affirms that it is in large part a unitary disorder. Our model helps explain why feline stress responses tend to be somaticized most dramatically in the urogenital tract, since it implies that part of the stress is usually psychosexual in origin. Involvement of adrenal androgens would not only take some of the mystery out of the 5 “urias” and urethral spasm–which are omnipresent in idiopathic cases–but also cases involving urolithiasis and urethral plugs, as outlined diagramatically above.

Whether due to stress from external causes or to an endogenous physiological process, we believe that FUS may result as an outcome of the dual role of AA as partial agonists of the androgen receptor mediating sexual behavior and as an adaptation to chronic stress in certain feline patients. (see Figure 2A). In that light, rising androgen levels in late winter and spring may constitute a “perfect storm” of factors that lead to an increased incidence of complete urinary obstruction. Androgens may rise not only because of chronic stress in FUS individuals, but breeding season itself may be the underlying stress leading to postulated adaptive response.

In particular, we contend that at least part of the “stress” experienced by the predisposed cats is psychosexual in origin. Androgens are known to heighten sexual arousal in both males and females (20), and neutering may create a state of suspended arousal, due to inability to complete sex-typical behaviors, due to absence of either testosterone or estrogen.

Perhaps dovetailing with other environmental stresses [e.g., earthquakes in Caston's study 14), psychosexual stress leads to irritative voiding (21, 22). This most straightforwardly explains the idiopathic FUS or FIC cases. In addition, however, rising AA may also explain many of the cases where an obvious proximate cause is found including urolithiasis, and mixed mucus and mineral urethral plugs. Androgens are well known to drive mucus production by bulbourethral glands (23, 24), in preparation for ejaculation, which does not occur in absence of testosterone in neutered cats, and this likely contributes to formation of mucus plugs.

Regarding urolithiasis, it is known that men are three times more likely to develop renal stones than are women (25). Although most studies link greater susceptibility of men to uroliths to testosterone, as in rats (26), not all do. In men, it may be that AA play a role as well, as this would help explain why risk of urolithiasis is so much greater in men but not always in direct correlation to plasma testosterone (27). So it seems plausible that adrenal androgens may heighten susceptibility to urolithiasis in cats. In addition, as described elsewhere, neutering alters the composition of the extracellular matrix of penile tissues, likely making it less distensible, and more prone to lodging of uroliths (28) (see Figure 2B).

## Risk factors for FUS in light of the adrenal androgen model

Based on our model, neutering is suggested to be a major risk factor because testosterone seems to have such powerful effects on normal feedback inhibition of the hypothalamic-pituitary-adrenal axis *in toto* (29). Loss of testosterone likely has multiple physiological and clinical effects including: (1) loss of feedback inhibition of stress hormones like corticosterone from the H-P-A axis not only leads to greatly enhanced stress responses in male cats, as shown for rats (30), but also increased adrenal androgen production (31)—particularly during late winter/spring (32), (2) during that time, insufficient “release” of the build-up of hormonal stress/tension through execution of appropriate sex-determined behaviors, and possibly (3) increased androgen receptor (AR) concentration on the surface of hormone-responsive cells both in the hypothalamus and urogenital tract (33) which may “prime” the cats for explosive responses when adrenal androgen putatively rise in late winter/spring (34). Loss of feedback inhibition on adrenal steroid production may be a shared pathogenetic feature of urethral obstruction in neutered cats and ferrets, which suggests that ferrets may provide at least a partial model for cats (35).

What other FUS risk factors might make sense in light of the AA hypothesis? One significant susceptibility factor that has been repeatedly shown in epidemiological studies is body weight. Although most authors have construed this as related to obesity and sedentary lifestyle (36), as originally pointed out by Willeberg, “the correlation between actual weight and adiposity in cats cannot be considered simple, as it may be due to breed, sex, age, *and individual variation in body frame*.” (Emphasis added) (6).

**Figure 3:**
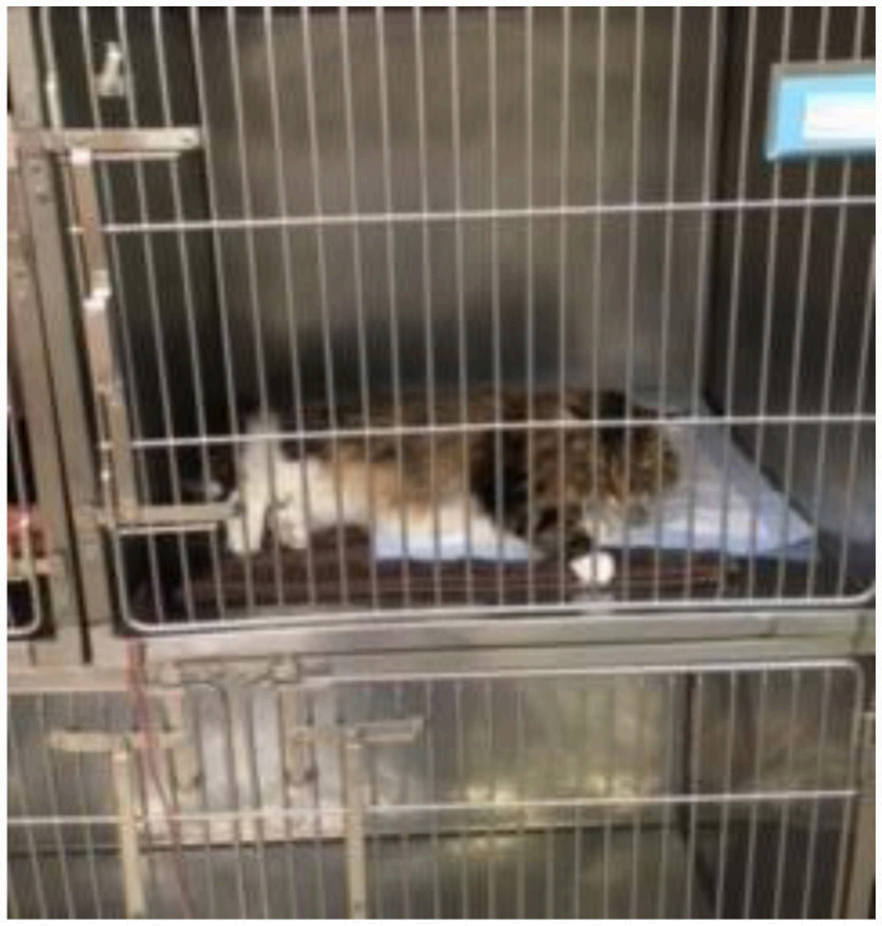
FUS risk factors through the lens of the AA model: is body weight or body frame size the real factor? (Photograph courtesy Dr. Steven Findlay, St. Francis Animal Hospital). Viewed through the lens of the AA model, risk factors for FUS take on slightly new meanings. For instance, many epidemiological studies have determined that high body weight (>5.8 kg) is a risk factor. Although this has often been interpreted to reflect excess adiposity and sedentary lifestyle, this has never been proven. It is just as likely that the high weights are a function of larger than average *body frame size*. This in turn, may be determined by total androgen exposure during development, which, along with neutering, may make very large male cats exceptionally vulnerable to later FUS and urethral obstruction, perhaps due to higher concentrations of androgen receptors in cells of urogenital tissues.

One observation repeatedly made by both authors in practice in western Pennsylvania is that most neutered male “blocked” cats have very large body frame size, with or without excess adiposity. A photograph of a cat seen for urethral obstruction at St. Francis Animal Hospital on February 20, 2018 is fairly typical (see Figure 3). He was a 7 years old Maine Coon cat whose body weight was 9.7 kg, but with fairly normal BCS of approximately 5/9. He was 27 cm high at the withers, and 43 cm long from base of neck to base of tail.

As androgens are one of the main determinants of adult male mammal body size (37), it may be that increased AA levels during development of young neutered cats may compensate for lack of testosterone while at the same time heightening risk for later FUS and obstruction. It may be that larger male cats have a more prolonged period of adrenal androgen exposure as they grow and/or higher levels of androgens. This may somehow “set the stage” for more severe stress reactions to adrenal androgen surges as day length increases, perhaps because these big cats have much higher concentrations of AR on surfaces of tissues of their urogenital tracts and perhaps their hypothalami. There is good evidence that AA do partly compensate for absent testosterone in neutered male cats, as urethral diameters in early-age neutered cats acquire the same size as in intact cats (although recent findings show castrated cats have abnormal ECM in their penile tissues). In contrast to expectations, all studies to date suggest that development of the urethra in male cats is not dependent on presence of normal concentrations of testosterone (38, 39). However, it may be that adrenal androgens compensate either by themselves acting on target tissues or by producing testosterone in those tissues indirectly.

The zona reticularis of the adrenal gland itself produces a small amount of testosterone while predominantly secreting androgens such as dehydroepiandrosterone, or DHEA, and DHEA sulfate, or DHEA-S, into the blood (40). Compared to testosterone, DHEA and DHEA-S bind less efficiently to the androgen receptors that convert hormone signals into cellular results. However, some target tissues for DHEA and DHEA-S convert those weak androgens to testosterone (41), amplifying the strength of the cellular response. So neutered cats have adrenal glands that produce adrenal androgens, and indirectly, a small amount of testosterone (32). Although adrenal androgens like DHEA and DHEA-S are considered “weak,” their actual actions *in vivo* in neutered adult cats are essentially unknown. Although AA interact with AR less well than testosterone, there are definitely mechanisms of androgen triggering of cells that are not clearly receptor mediated. AA may operate partly through these alternative mechanisms (42).

## Testing the adrenal androgen (AA) model of FUS: direct vs. indirect approaches

First and foremost, we need an assay for all feline adrenal androgens. The University of Tennessee endocrinology service does measure androstenedione, which is an adrenal androgen in neutered cats. But there is currently no test for DHEA or DHEA-S. A complete test of the AA model would involve attempting to answer a wide range of questions, from very basic to clinical. As very little is known about AA dynamics in any species, except perhaps for human beings (41), the variety of feline studies that might be undertaken is practically unlimited. However, we have had a few relatively straightforward ideas for testing. The two main approaches we have considered are to: (1) probe AA and AR involvement directly in cats, or a more indirect approach, (2) to design an intervention aimed at blocking AA and to see if that alters the usually natural history of FUS, particularly with obstruction.

For the first approach, measurements of AA levels during life and perhaps also AR levels on both urogenital tract and central H-P-A tissues postmortem are very much needed in chronic FUS cats vs. controls, and also in blocked cats that re-obstruct vs. those that remain FUS free. Obstruction is a particularly useful endpoint, because it is easily determined by objective means (i.e., inability to urinate). As re-obstruction rates (rUO) vary widely from 15–40% in published series, it would be very helpful for all large practice and university-based veterinary hospitals to determine their rUOs following successful short-term management. This would greatly facilitate power calculations to determine the number of cats needed to conduct a statistically-meaningful test of correlation between AA and/or AR levels and likelihood of re-obstruction.

For an indirect test of the AA model, it would make sense to design a treatment based on the model, and see if it works. Again, a focus on obstruction would provide the most objective test. The current standard post-obstruction protocol relies mainly on one controlled study. Compared to phenoxybenzamine, Hetrick showed that the alpha-1 antagonist prazosin lowered the rUO at 24 h to 7.14% (vs. 21.74%) and at 30 days 18.18% (vs. 39.02%) (43). Instead, or perhaps in addition to that protocol, we have strongly considered whether deslorelin implants might be effective in preventing re-obstruction. A GnRH agonist, deslorelin, would be a reasonable first candidate because of its demonstrated safety and efficacy in fertility control and treatment of certain hormonal disorders of cats (44). In ferrets, deslorelin implants have been very effective in suppressing adrenal steroid production for about a year. In cats, the suppression of adrenal steroid production lasts for 20 months in males, and 24 months in females (45).

## Discussion and conclusions: toward simpler management of feline urinary disturbances

Most of what is known about effects of adrenal androgens (AA) derives from human studies. It is known that, in situations of low/absent testosterone, AA definitely induce well-known masculine characteristics in women and prepubescent boys and girls. For instance, in prepubescent girls, andrenal androgens cause growth of pubic and axillary hair (“andrenarche”) (46). It may, therefore, be that, in early neutered cats, AA are behind the capacity for normal development of urogenital structures. But the downside may be that AA cannot fully compensate for absent testosterone, FUS being the result.

Much of past debate about classifying feline lower urinary tract problems is due to two conflicting tendencies in model-building: (1) to treat each individual case as a separate entity according to anatomic site affected and presence/absence of an etiologic agent (proximate focus) (2) or (2) to attempt to identify a common feature that somehow unifies the various cases under one main etiologic umbrella (ultimate focus) (6). Practitioners have often felt buffeted between these two extremes, leading to uncertainties about appropriate management. It is very difficult for us as practitioners to integrate our understanding of concrete proximate causes of feline lower urinary tract disease (FLUTD) with the somewhat airy concept that FLUTD is caused by all manner of stress (FIC). It is our hope that in providing a more solid endocrinologic basis for FUS that practitioners may be able to simplify their understanding and explanations of feline urinary disturbances, and that further research based on our model may improve treatment and prevention of FUS including obstruction.

## Author contributions

BR conceived of the adrenal androgen hypothesis of FUS including postulated mechanistic detail and did the bulk of writing. RW provided input on how to test the idea either directly, or indirectly, based on prior work in ferrets.

### Conflict of interest statement

The authors declare that the research was conducted in the absence of any commercial or financial relationships that could be construed as a potential conflict of interest.
